# Blood Pressure in Patients Attending an Out-of-Hours Dental Clinic

**DOI:** 10.7759/cureus.92878

**Published:** 2025-09-21

**Authors:** Yosuke Iijima, Miki Yamada, Nami Nakayama, Takumi Takahashi, Shunsuke Hino, Motohiko Sano, Norio Horie, Takahiro Kaneko

**Affiliations:** 1 Department of Oral and Maxillofacial Surgery, Saitama Medical Center, Saitama Medical University, Saitama, JPN; 2 Division of Applied Pharmaceutical Education and Research, Hoshi University, Tokyo, JPN

**Keywords:** high blood pressure, hypertension and diabetes, normal blood pressure, out-of-hours patient care, potential hypertension, urgent dental care

## Abstract

Objective: This study aimed to evaluate blood pressure in patients attending an out-of-hours dental clinic, including oral surgery treatment, and compare it with the 2019 National Health and Nutrition Survey (National Survey) in Japan. The prevalence of treated hypertension and diabetes was also assessed.

Materials and methods: A retrospective study was conducted on patients aged 20 years or older who visited the out-of-hours dental clinic between February 19, 2013, and May 8, 2024. Systolic blood pressure (SBP), diastolic blood pressure (DBP), and the prevalence of treated hypertension and diabetes were compared with the National Survey. Fisher’s exact test was used for categorical variables where appropriate. All statistical tests were two-sided, and p-values < 0.05 were considered statistically significant. Statistical analysis was performed using js-STAR software v9 (www.kisnet.or.jp/nappa/software/star/index.htm; developed by Satoshi Tanaka and Hiroyuki Nakano, Japan).

Results: A total of 2511 out-of-hours dental clinic outpatients were included in the study. The distribution was 1344 (53.5%) male and 1167 (46.5%) female patients, with a gender ratio of 1.2:1. Blood pressure was significantly higher in out-of-hours dental clinic patients compared to the National Survey (p < 0.001), except for specific age/gender groups. The prevalence of treated hypertension and diabetes was not significantly different between the two groups (p > 0.05), except for certain subgroups.

Conclusion: The out-of-hours dental clinic patients exhibited significantly higher blood pressure than the general population, suggesting a need for careful monitoring. The comparable prevalence of treated hypertension and diabetes indicates potential undiagnosed cases, highlighting the importance of systemic health assessments in out-of-hours dental care.

## Introduction

Blood pressure is a fundamental vital sign, and a significant relationship exists between hypertension and dental health. Dental procedures can induce physiological stress responses, including elevated blood pressure, particularly during painful treatments without local anesthesia [1]. Dental visits are inherently stressful, yet comprehensive blood pressure studies in dental patients remain scarce [2-4]. Kimura et al. measured the blood pressure of patients who had a first dental visit at different ages and linked the increase in blood pressure to stress [2].

Saitama Medical Center at Saitama Medical University, Saitama, Japan, is a regional core hospital and is affiliated with the Advanced Center for Emergency Medicine and Critical Care. Our department, dealing with dental and oral surgery patients, provides both regular daytime and evening out-of-hours care. A large number of patients visit the out-of-hours clinic with a sense of anxiety because of the urgent nature of the treatment and the fact that it is at night. Clinical observation indicates that patients presenting to out-of-hours clinics often exhibit elevated blood pressure. This suggests that these patients may experience higher stress levels compared to those attending regular dental appointments, potentially leading to greater blood pressure increases. Exaggerated cardiovascular reactions to acute psychological stress may contribute to the etiology of cardiovascular pathology, posing particular risks for hypertensive patients undergoing dental procedures [5,6].

In Japan, the 2019 National Health and Nutrition Survey (National Survey) serves as a survey to understand the population's physical condition, nutrient intake, and lifestyle habits, providing essential data for promoting public health under the Health Promotion Act. The National Survey examines dietary habits, blood pressure levels, hypertension, diabetes prevalence, and various physical and blood tests, as well as alcohol consumption, smoking, and exercise habits. It is an indispensable survey for national health promotion measures and lifestyle-related disease countermeasures [7]. It is widely used as a comparative benchmark for various studies conducted in Japan. It should be added that hypertension and diabetes mellitus are among the most common conditions for which patients seek consultation with internal medicine specialists in Japan, and individuals with diabetes often have hypertension as a complication. However, detailed reports on the prevalence of hypertension and diabetes among dental patients are scarce [8,9].

This study aimed to investigate whether the blood pressure of patients attending an out-of-hours dental clinic increased compared to the 2019 National Survey in Japan. Additionally, the study sought to determine the prevalence of known hypertension and diabetes mellitus cases in the same population.

## Materials and methods

This retrospective study was approved by the Research Ethics Committee of Saitama Medical Center, Saitama Medical University (approval number: SOU2025-033).

Study population

Patients aged 20 years or older who visited the out-of-hours oral surgery clinic at Saitama Medical Center between February 19, 2013, and May 8, 2024, were included. The out-of-hours outpatient clinic operates from 17:30 to 08:30 hours the following day. 

Exclusion criteria

Patients were excluded if their medical records lacked sufficient documentation or if they were seen by an oral surgeon for conditions unrelated to dental or oral surgery, such as those admitted to the ICU or emergency department. In cases where a patient visited the clinic multiple times, only data from the first visit were included. 

Data collection

The following data were collected: systolic blood pressure (SBP) and diastolic blood pressure (DBP) at presentation, and the presence of treated hypertension and diabetes mellitus. It is important to note that only patients currently under treatment for hypertension and diabetes were included. This is because, for various reasons, some individuals diagnosed with hypertension or diabetes do not undergo treatment, and it was not possible to reliably identify untreated cases. Blood pressure measurements were taken by nurses in the after-hours consultation room using either electronic or manual sphygmomanometers. Measurements were taken as soon as possible after the patient's arrival, with patients seated or, if unable to sit, lying in bed. Due to consultation priorities, only one measurement was taken. Additionally, in the case of the National Survey, blood pressure measurements were taken using manual sphygmomanometers in a seated position after a five-minute rest period. The blood pressure value used was the average of two measurements. For those for whom only one measurement could be taken, that single value was adopted. 

Data analysis

The collected data were categorized by gender and age groups (20s, 30s, 40s, 50s, 60s, and 70s or older) and compared with data from the 2019 National Survey in Japan. 

Statistical analysis

Data were obtained from the National Survey 2019. Gender and age distributions, as well as systemic factors including hypertension and diabetes, were compared between the study and control groups. Fisher’s exact test was used for categorical variables where appropriate. All statistical tests were two-sided, and p-values < 0.05 were considered statistically significant. Statistical analysis was performed using js-STAR software v9 (www.kisnet.or.jp/nappa/software/star/index.htm; developed by Satoshi Tanaka and Hiroyuki Nakano, Japan).

## Results

A total of 2511 out-of-hours clinic outpatients were included in the study, comprising 1344/2511 (53.5%) male patients and 1167/2511 (46.5%) female patients, with a male-to-female ratio of 1.2:1. The age distribution of the participants is detailed in Table 1.

**Table 1:** Age distribution of patients by sex

Sex	Age (y)						Total
	20 - 29	30 - 39	40 - 49	50 - 59	60 - 69	≥ 70	
Male (n)	289 (20.50%)	198 (14.73%)	228 (16.96%)	167 (12.43%)	152 (11.31%)	310 (23.07%)	1344
Female (n)	176 (15.08%)	140 (12.00%)	185 (15.85％)	148 (12.68%)	145 (12.43%)	373 (31.96%)	1167

Blood pressure measurements

The results of blood pressure measurements are presented in Table 2. SBP ± standard deviation (SD) in male patients was 125.0 ± 14.7 mmHg in their 20s, and it increased with age up to the 60s, before starting to decline from their 70s onwards. DBP increased from the 20s (73.6 ± 12.8 mmHg) to the 50s, then began to decrease after the 60s. A similar trend was observed in the National Survey, with SBP rising from the 20s (115.3 ± 13.7 mmHg) to the 60s, then plateauing from the 70s onwards. DBP also increased from the 20s (67.7 ± 9.3 mmHg) to the 50s, before declining after the 60s. In female patients, the results of the present study showed that SBP ± SD increased from the 20s (118.4 ± 13.7 mmHg) to the 70s. DBP rose from the 20s (72.8 ± 11.1 mmHg) to the 50s and then began to decline after the 60s. Similarly, in the National Survey, SBP increased from the 20s (105.7 ± 10.1 mmHg) to the 70s. DBP varied from the 20s (66.3 ± 7.7 mm Hg) onwards and was the highest in the 60s (Table 2). A comparison of blood pressure values between the present study and the National Survey revealed significantly higher SBP and DBP in the out-of-hours clinic patients across all age groups, except for SBP in male patients in their 40s (p = 0.736) and DBP in female patients in their 30s (p = 0.378) (Table 2). 

**Table 2:** Comparison of blood pressure measurements between out-of-hours dental clinic patients in the present study and the 2019 National Survey. SBP, systolic blood pressure; DBP, diastolic blood pressure; SD, standard deviation; National Survey, National Health and Nutrition Survey in Japan, 2019 [7]

Parameter		Age (y)					
		20 - 29	30 - 39	40 - 49	50 - 59	60 - 69	≥ 70
Male, Present study	SBP (mean ± SD mmHg)	125.0 ± 14.7	126.6 ± 16.9	126.5 ± 18.8	142.1 ± 21.4	146.7 ± 24.4	145.5 ± 24.2
	DBP (mean ± SD mmHg)	73.6 ± 12.8	77.2 ± 13.5	85.5 ± 14.2	88.7 ± 16.2	86.2 ± 15.3	81.6 ± 15.6
Male, National Survey	SBP (mean ± SD mmHg)	115.3 ± 13.7	117.3 ± 11.7	125.8 ± 16.0	131.7 ± 18.4	135.8 ± 18.1	135.8 ± 16.1
	DBP (mean ± SD mmHg)	67.7 ± 9.3	73.7 ± 10.3	81.3 ± 12.2	82.0 ± 11.7	78.5 ± 10.4	73.1 ± 10.3
p-value (Present study vs. National Survey)	SBP	p < 0.001	p < 0.001	p = 0.736	p < 0.001	p < 0.001	p < 0.001
	DBP	p = 0.002	p = 0.093	p = 0.013	p = 0.001	p < 0.001	p < 0.001
Female, Present study	SBP (mean ± SD mmHg)	118.4 ± 13.7	121.0 ± 15.9	131.8 ± 23.0	138.5 ± 21.9	147.0 ± 23.9	149.7 ± 25.4
	DBP (mean ± SD mmHg)	72.8 ± 11.1	75.0 ± 13.9	78.5 ± 16.3	82.6 ± 13.7	82.4 ± 13.7	80.1± 15.8
Female, National Survey	SBP (mean ± SD mmHg)	105.7 ± 10.1	107.9 ± 13.1	114.3 ± 15.9	123.7 ± 18.4	131.0 ± 16.0	136.1 ± 16.9
	DBP (mean ± SD mmHg)	66.3 ± 7.7	73.7 ± 9.6	71.2 ± 10.7	75.4 ± 11.7	76.7 ± 10.1	73.0 ± 10.3
p-value (Present study vs. National Survey)	SBP	p < 0.001	p < 0.001	p < 0.001	p < 0.001	p < 0.001	p < 0.001
	DBP	p < 0.001	p = 0.378	p < 0.001	p < 0.001	p < 0.001	p < 0.001

Prevalence of treated hypertension

The proportion of individuals receiving treatment for hypertension in the present study was 1/289 (0.35%) among male patients in their 20s, increasing to 171/310 (55.16%) in their 70s and older. Among female patients, the rate was 0/176 (0.00%) in their 20s and increased to 184/373 (49.33%) in their 70s and beyond. These values for male and female patients were generally lower than the proportion of individuals with hypertension, including those not receiving treatment, reported in the National Survey. However, a comparison of the values in this study with the proportion of those receiving treatment for hypertension in the National Survey revealed no significant differences at any age for either sex, except for female patients in their 30s (Table 3).

**Table 3:** Comparison of hypertension prevalence between out-of-hours dental clinic patients in the present study and the 2019 National Survey. National Survey, National Health and Nutrition Survey in Japan, 2019 [7]

Parameter	Age (y)					
	20 - 29	30 - 39	40 - 49	50 - 59	60 - 69	≥ 70
Male, Present study	1/289 (0.35%)	4/198 (2.02%)	31/228 (13.6%)	43/167 (25.75%)	68/152 (44.74%)	171/310 (55.16%)
Male, National Survey	1/55 (1.82%)	1/62 (1.61%)	14/122 (11.48%)	35/132 (26.52%)	123/265 (46.42%)	237/443 (53.5%)
p-value (Present study vs. National Survey)	p = 0.4307	p = 0.1876	p = 0.2986	p = 0.8697	p = 0.7426	p = 0.4899
Female, Present study	0/176 (0.00%)	5/140 (3.57%)	15/185 (8.11%)	21/148 (14.19%)	47/145 (32.41%)	184/373 (49.33%)
Female, National Survey	0/53 (0.00%)	0/119 (0.00%)	8/220 (3.64%)	29/232 (12.5%)	97/351 (27.63%)	263/523 (50.29%)
p-value (Present study vs. National Survey)	p = 1.000	p = 0.0143	p = 0.2986	p = 0.69	p = 0.21	p = 0.82

Prevalence of treated diabetes

The proportion of individuals receiving treatment for diabetes in the present study was 1/289 (0.35%) among male patients in their 20s, increasing to 59/310 (19.03%) in their 70s and older. Among female patients, the proportion was 4/176 (2.27%) in their 20s, then varied across age groups but generally increased, reaching 55/373 (14.75%) in their 70s and older. These values for male and female patients with diabetes were lower than the proportion of individuals with diabetes, including those not receiving treatment, reported in the National Survey, at any age, except for female patients in their 20s. However, there were no significant differences between those receiving diabetes treatment in this study and the National Survey at any age for either sex, except for female patients in their 20s (Table 4).

**Table 4:** Comparison of diabetes mellitus prevalence between out-of-hours dental clinic patients in the present study and the 2019 National Survey. National Survey, National Health and Nutrition Survey in Japan, 2019 [7]

Parameter	Age (y)					
	20 - 29	30 - 39	40 - 49	50 - 59	60 - 69	≥ 70
Male, Present study	1/289 (0.35%)	1/198 (0.51%)	9/228 (3.95%)	11/167 (6.59%)	27/152 (17.76%)	59/310 (19.03%)
Male, National Survey	0/55 (0%)	0/64 (0%)	4/115 (3.48%)	17/129 (13.18%)	56/249 (22.49%)	80/401 (20.0%)
p-value (Present study vs. National Survey)	p = 0.3541	p = 0.1706	p = 0.1706	p = 0.1706	p = 0.0610	p = 0.6845
Female, Present study	4/176 (2.27%)	5/140 (3.57%)	4/185 (2.16%)	12/148 (8.11%)	16/145 (11.03%)	55/373 (14.75%)
Female, National Survey	0/45 (0%)	3/114 (2.63)%	2/211 (0.95%)	11/221 (4.98%)	29/328 (8.84%)	68/480 (14.17%)
p-value (Present study vs. National Survey)	p = 0.0435	p = 0.4497	p = 0.4497	p = 0.6389	p = 0.0945	p = 0.6989

## Discussion

This study indicates that patients attending an out-of-hours dental clinic have significantly higher blood pressure compared to the general population. Furthermore, the prevalence of hypertension and diabetes during treatment is comparable, suggesting the potential existence of undiagnosed cases.

According to the 2019 National Survey, the mean SBP in the Japanese population over the past decade was 132.0 mmHg in male patients and 126.5 mmHg in female patients, with a significant decrease observed in both sexes. The proportion of individuals with SBP of 140 mmHg or higher also significantly decreased, with 29.9% of male and 24.9% of female patients affected [7]. Despite this decline, the prevalence of hypertension remains high in Japan, potentially due to inadequate treatment and control rates, as well as factors like obesity, alcohol intake, and diet [10-12].

Regarding the relationship between stress and blood pressure, a meta-analysis indicated that individuals with stronger stress responses were 21% more likely to experience blood pressure increases [13]. Acute stress elevates blood pressure by increasing cardiac output and heart rate, along with levels of catecholamines and cortisol [14]. Dental visits and treatments have long been recognized as stressful, inducing physiological stress responses such as increased blood pressure, elevated cortisol levels, and heightened sympathetic nervous system activity [1,15-17]. Studies have also reported that dental patients exhibit higher blood pressure than medical patients [18].

While previous studies have examined blood pressure in first-time dental outpatients, statistical observations of out-of-hours dental clinic outpatients are lacking [2]. Kimura et al. compared first-time dental patients with the 2012 National Survey and reported significantly higher SBP in younger patients (20s-40s for men, 20s-50s for women) compared to the National Survey. No significant differences were found in older age groups or in DBP. They stated that high SBP in relatively young patients may be related to psychosocial backgrounds as well as medical causes [2]. The blood pressure in this report was compared with the blood pressure of Japanese individuals of different age groups (including users of antihypertensive drugs) from the National Survey 2019. SBP and DBP for both male and female patients were significantly higher than those of the National Survey in both age groups, although there was no significant difference between SBP in male patients in their 40s and DBP in female patients in their 30s. This may be attributed to the stressful nature of emergency dental visits, the time of day, or underlying health conditions. Notably, our results also showed higher SBP and DBP values compared to first-time dental patients reported by Kimura et al., except for SBP in male patients aged 20s to 40s [2]. 

When measuring blood pressure in an outpatient setting, white coat hypertension should be considered [19]. Characterized by elevated blood pressure during medical visits but normal ambulatory readings, white coat hypertension is a stress-induced response involving acute stress and sympathetic activation [20]. With prevalence rates exceeding 45% in some age groups [21], it is plausible that the elevated blood pressure observed in our study's out-of-hours dental clinic patients is partly attributable to white coat hypertension. The elevated blood pressure due to the stress of dental treatment and the elevated blood pressure due to white coat hypertension are both due to stress and cannot be distinguished. Regardless, our findings suggest that patients visiting an out-of-hours dental clinic exhibit higher blood pressure than the general population and first-time dental patients. 

In terms of hypertension prevalence, Miyawaki et al. did not specify criteria or confirmation methods for hypertension [9]. However, they stated that the prevalence of hypertension among Japanese dental patients was 10.9% and that the blood pressure readings of dental patients were similar to those in National Surveys, reflecting the prevalence of hypertension. Our study focused on patients receiving medication, resulting in lower values compared to the overall prevalence in the National Survey 2019. However, no significant difference was found compared to the proportion of treated hypertensive individuals in the National Survey, except for female patients in their 30s. This suggests that a substantial number of poorly treated hypertensive patients visit out-of-hours dental clinics. 

Diabetes was included in this study due to its close association with clinical dentistry and the bidirectional relationship between diabetes and oral health [22]. According to the 2019 National Survey, the proportion of individuals strongly suspected of having diabetes was 19.7% for men and 10.8% for women. Over the past decade, no significant increase or decrease has been observed for either gender. When analyzed by age group, the proportion is higher among older age groups [7]. Studies have reported a prevalence of 1.7-46.4% for type 2 diabetes and 23.3-68.0% for prediabetes among dental patients denying hyperglycemia [21]. Similar to hypertension, the values in our study were not significantly different from the percentage of National Survey diabetes patients actually receiving treatment, for both male and female patients in any age group, except for female patients in their 20s. This suggests the presence of numerous undiagnosed diabetic patients among out-of-hours dental clinic visitors. Given that 90% of diabetic individuals are unaware of their condition, early detection is crucial [21]. The contribution of dental practitioners through screening and periodontal assessment was noted in the detection of diabetes, and close examination of highly inflammatory and excessively hypertensive patients, which are common in out-of-hours dental outpatient clinics, could help to find patients with untreated diabetes and hypertension, respectively [23].

This study has several limitations. First, this study was conducted at a single institute. Hence, the number of patients is limited. The lack of significant differences in the values of some hypertension and diabetes patient groups in this study compared to the National Survey may be due to the small sample size. Second, in this study, patients with hypertension and diabetes were restricted to those currently receiving treatment. However, careful consideration of medical and medication histories may have aided in identifying a broader range of patients with hypertension and diabetes. Third, blood pressure measurements were taken in a busy emergency center, not in a calm resting state as in the National Survey, potentially introducing measurement environment bias. Fourth, our hospital is a regional core hospital with an advanced emergency center, possibly attracting more severely ill patients who might exhibit higher blood pressure. Fifth, the study included all out-of-hours patients collectively. Disease-specific analyses, such as focusing on patients with severe inflammation, might reveal higher diabetes prevalence.

## Conclusions

This study demonstrates that patients visiting an out-of-hours dental clinic exhibit significantly higher blood pressure compared to the general population, suggesting a need for careful monitoring and management of blood pressure in this patient group. Furthermore, the prevalence of treated hypertension and diabetes was comparable to the 2019 National Survey, indicating a potential for undiagnosed cases. This study provides a valuable database for future research in dental and oral surgery and highlights the importance of incorporating systemic health assessments in out-of-hours dental care.
